# Neuroprotective agents in the management of glaucoma

**DOI:** 10.1038/s41433-018-0050-2

**Published:** 2018-02-23

**Authors:** C. Nucci, A. Martucci, C. Giannini, L. A. Morrone, G. Bagetta, R. Mancino

**Affiliations:** 1 Ophthalmology Unit, Department of Experimental Medicine and Surgery, University of Rome Tor Vergata, Rome, Italy; 2 Department of Pharmacy, Health and Nutritional Sciences, University of Calabria, Rende, Italy

**Keywords:** Drug therapy, Optic nerve diseases

## Abstract

Glaucoma is an optic neuropathy, specifically a neurodegenerative disease characterized by loss of retinal ganglion cells (RGCs) and their axons. The pathogenesis of RGC loss in glaucoma remains incompletely understood and a broad range of possible mechanisms have been implicated. Clinical evidence indicates that lowering intraocular pressure (IOP) does not prevent progression in all patients; therefore, risk factors other than those related to IOP are involved in the disease. The need for alternative, non-IOP-lowering treatments focused at preventing progression, that is, neuroprotectants, has become of interest to both the patient and the physician. Experimental evidence accumulated during the past two decades lend a great deal of support to molecules endowed with neuroprotective features. However, translation to the clinic of the latter drugs results unsuccessful mostly because of the lack of reliable in vivo measure of retinal damage, thus hampering the good therapeutic potential of neuroprotective agents given alone or as adjuvant therapy to IOP-lowering agents. Further research effort is needed to better understand the mechanisms involved in glaucoma and the means to translate into clinic neuroprotective drugs.

## Introduction

Glaucoma is a progressive optic neuropathy characterized by loss of retinal ganglion cells (RGCs) and typical visual field defects. With more than 60 million people affected, it is nowadays considered a leading cause of irreversible blindness worldwide [1]. Ocular hypertension has been proven to be an important risk factor involved in the onset and progression of the disease [2]. Nonetheless, international clinical trials have shown that in some patients under conditions of intraocular pressure (IOP) lowering the disease develops and progresses. These data have also been confirmed by recent studies conducted on patients treated and monitored during their lifetime in highly specialized glaucoma centers [3, 4]. The analysis of the clinical record of a sample of 592 subjects with glaucoma who died between 2006 and 2010 showed that in the last visit 42.2% of them were blind in one eye and 16.4% were blind bilaterally. These data support the hypothesis that risk factors other than IOP intervene in the pathogenesis of the neuronal damage in glaucoma.

Recent experimental studies show that the disease-induced damage is not limited to the retinal and axon fibers of the optic nerve but also extends to the brain. The latter evidence was initially observed in animal experimental models, and then, owing to the use of advanced neuroimaging techniques, confirmed in humans, highlighting a connection between eye damage and alterations in central visual pathways [2, 5, 6].

The involvement of the central nervous system in glaucoma has been extensively proved in animal models, and because of the anatomical and functional similarities of the humans and primates visual pathways, researches have been primarily conducted in monkeys.

The first study on primate experimental glaucoma dates back to 2000. Weber et al. [7], using a glaucoma model in primates, documented a reduction of the number of neurons and their volume in the regions of the lateral geniculate nucleus connected with the affected eye. In this same model, IOP elevation caused a preferential degenerative effect on magnocellular regions rather than parvocellular regions of the geniculate nucleus. Neuronal loss, both in the magnocellular and parvocellular layers of the lateral geniculate nucleus connected with the primary visual cortex, was subsequently confirmed immunohistochemically [8, 9]. Interestingly, these same alterations described in the animal model have also been reported in humans. Chaturvedi et al. [10] were the first to examine autopsy sections of the lateral geniculate nucleus in individuals with and without glaucoma; the magnocellular cell density was significantly lower in the glaucoma group compared to the control group, although this did not occur in the parvocellular layer.

Histological evaluation of the intracranial portions of the optic nerves and of the central visual areas from a patient with glaucoma, who died for viral myocarditis, showed a pronounced atrophy of the optic nerve, the lateral geniculate nucleus, and visual cortex with respect to corresponding areas obtained from autoptic control samples [11]. Magnetic resonance imaging (1.5 Tesla) showed a volume reduction of the lateral geniculate nucleus also documented at the level of the histological sections comprising this structure [11, 12]. Dai et al. [13] subsequently confirmed these same anatomical alterations in 26 glaucoma patients using 3 Tesla magnetic resonance imaging. Moreover, in the latter study, the height and volume of the lateral geniculate nucleus, manually measured by two radiologists, were compared and correlated with the stage of the disease, derived from the visual field results, underscoring an inverse correlation between these parameters.

To overcome the limitations of conventional radiological methods and the bias of manual measure of the size of the lateral geniculate nucleus, brain involvement in glaucoma was studied by our group using 3 Tesla magnetic resonance imaging with diffusion tensor [14]. The diffusion tensor provides a reconstruction of the axonal architecture on the basis of diffusion of water molecules along the axons. Owing to the fiber tractography, this imaging technique provides colorimetric maps and allows two-dimensional or three-dimensional reconstructions of the examined fibers pathway. The optical nerve examination performed in three regions of interest, at different distances from the bulb, showed a close correlation between the stage of the disease and the numeric parameters of the diffusion tensor. Thus, the progression of the disease corresponded to an increase in mean diffusivity (MD) and a reduction in fractional anisotropy (FA) values. In this study, differences among control subjects and patients at stage 0 of glaucoma were not detected for both FA and MD values [14]. The usefulness of this radiologic technique has been validated further by a subsequent study in which these same parameters, evaluated 5 mm behind the eyeball, showed a good correlation with morphological aspects of the optic nerve head (ONH) and the thickness of the retinal nerve fiber layer assessed by GDx-VCC, Heidelberg Retina Tomography III, and Optical Coherence Tomography (OCT) [15]. Altogether, the data suggest that diffusion tensor parameters may be considered possible biomarkers of retrobulbar affections. It has been suggested that the complementary use of the high sensitivity of FA and of the high specificity of MD at the proximal site may provide reliable indices for the identification of glaucomatous patients at early stages and may prove useful to evaluate the efficacy of new therapeutic strategies [16].

Altogether, these observations in conjunction with the knowledge that lowering IOP may not be sufficient in some eyes to prevent the development of glaucoma or the associated progressive vision loss form the rational basis for the current development of neuroprotection as therapeutic strategy for glaucoma. Such therapeutic intervention has the purpose of interfering with the molecular mechanisms causing neuronal damage, acting on targets that are not concerned with IOP control, although enhancing the survival of retinal cells. Pathological processes underscoring neuronal death in glaucoma have been the focus of intense research work worldwide. By observing the complexity of these mechanisms, numerous molecules have been identified that have the potential to block neurodegenerative events induced by glaucoma. Since the advent of the concept of neuroprotection, a lot of molecules have been tested with very low success rates in the translation from the laboratory to patients. Accordingly, more than 100 neuroprotective drug candidates have failed to demonstrate efficacy, acceptable safety, or patient benefit. Most of them, in fact, despite successful preclinical data, failed to pass most of the Phase 2 and virtually all the Phase 3 clinical trials. For instance, memantine, a non-competitive *N*-methyl-d-aspartate (NMDA) subtype of glutamate receptor antagonist, already in use in the treatment of Alzheimer’s disease, showed convincing neuroprotective effect in animal models of glaucoma. At variance with the latter, however, an impressive phase 3 randomized multicenter clinical trial lasting more than 5 years, with estimated cost over $100 million, did not reveal significant benefit for memantine treatment in preventing the progression of visual field loss in patients with glaucoma.

Similar outcome was yielded by brimonidine, a selective α-adrenergic receptor agonist approved for chronic treatment in glaucoma, indicated for reducing IOP in patients with open-angle glaucoma or ocular hypertension. Brimonidine reduces aqueous humor production and stimulates aqueous humor outflow through the uveoscleral pathway. Preclinical studies have shown a neuroprotective effect on retinal ganglionic cells in animal models of optic nerve injury relevant to glaucoma [17]. Moreover, the Low-Pressure Glaucoma Treatment Study has shown that, over a 30-month period, patients treated with brimonidine had a significantly lower rate of visual field defect progression compared to subjects treated with timolol (9 vs. 30%), thus supporting the dual action of the molecule [17]. However, the study and, therefore, neuroprotective efficacy in humans, were questioned in two Cochrane reviews [18, 19], suggesting the need for further studies.

These events are mostly caused by conceptual and methodological problems that make difficult the translation of preclinical results to clinical glaucoma practice. Due to the heterogeneity of the disease and the presence of comorbidities, there is no animal model that fully mimic the human disease. Furthermore, in most experimental studies, the neuroprotective agent is given at the time of or prior to injury, while in human trial the intervention comes after the diagnosis of the disease. In addition, ocular bioavailability is often difficult to predict in human. An additional central difference with preclinical methodologies is the outcome measure in the clinic. Animal studies mostly employ histopathologic endpoints to assess treatment efficacy, while for ethical reasons, clinical trials, use functional outcomes, which most often take months to show any change [20]. Bearing all this in mind, implementation of current perimetric, electrophysiological and OCT measurements [21], together with the advent of new methods to quantify in vivo the death of ganglion cells (see the detection of apoptosing retinal cell (DARC) technique [22]), and of new imaging techniques, such as diffusion tensor imaging [2], could give a positive boost to neuroprotective research.

Experimental studies have shown that ganglion cells death in glaucoma is an extremely complex process triggered by different molecular mechanisms. In this regard, a fundamental role seems to be carried out by the activation of the excitotoxic glutamate cascade resulting in excessive calcium intake within the cells. Such events may include alteration of the mitochondrial function, resulting in excess free radicals production and reduction of energy production. In addition, protein misfolding, oxidative stress, deprivation of neurotrophins and activation of inflammatory processes by the glial cells of the retina seem to play an important role [23, 24]. Therefore, it appears evident that there are multiple therapeutic targets on which neuroprotective molecules may act [25]. Below, we will discuss those that have been most studied and have more promising therapeutic perspectives.

Coenzyme Q10 (CoQ10) is an important component of the mitochondrial respiratory chain that plays a critical role in mitochondrial oxidative phosphorylation by serving as an electron carrier in the respiratory electron transport chain from complexes I and II to complex III, by which ATP is produced. By protecting lipids, proteins and DNA damaged by oxidative stress, CoQ10 plays a role of lipid-soluble antioxidant. Interestingly, CoQ10 supplementation therapy showed neuroprotective activity slowing, or reversing, pathological conditions including cerebral ischemia, Parkinson’s and Huntington’s diseases, and Leber hereditary optic neuropathy [26]. In addition to its role as an antioxidant, CoQ10, through the inhibition of mitochondrial depolarization by preventing the formation of the mitochondrial permeability transition pore (PTP), seems to protect against glutamate excitotoxicity in vivo.

Using an established animal model of retinal ischemia/reperfusion, it was shown that intraocular administration of CoQ10 reduces glutamate increase and affords neuroprotection, suggesting that oxidative stress and energy failure might be implicated in the mechanisms of RGC death [27]. In particular, it has been highlighted a role for abnormal elevation of extracellular glutamate in the mechanisms underlying RGC death that occurs, at least in part, via activation of the apoptotic program. The mechanism underlying cell loss involves overactivation of NMDA and non-NMDA glutamate receptors and consequent accumulation of nitric oxide (NO).

Using DARC imaging, Guo et al. [28] investigated the effects of topical CoQ10 on RGC apoptosis in vivo in a rat model of drug-induced RGC apoptosis using intravitreal staurosporine (SSP). The authors showed that the administration of CoQ10 0.1% significantly reduced SSP-induced RGC apoptosis compared to CoQ10 0.05% and carrier. The latter effect could be induced by inhibition of mitochondrial depolarization and transition pore opening, cytochrome *c* release, and caspase-9 activation [28].

Topical treatment solution of CoQ10 and vitamin E showed the ability of minimizing retinal damage and RGC loss also inhibiting the PTP formation and cytochrome *c*, induced by excessive activation of glutamate receptors, to be released from the mitochondrial intermembrane space into the cytosol, causing RGC death [29]. Another interesting possibility is that CoQ10 may reduce the accumulation of extracellular glutamate consequently reducing the detrimental action of ischemia/reperfusion on mitochondrial energy metabolism and on the function of glutamate transporters, thus preventing apoptotic death of RGC in rat [29]. This was also confirmed in a study that used both in vitro and in rodent mitochondrial-mediated neurotoxicity models to evaluate the neuroprotective activity of CoQ10/α-tocopherol polyethylene glycol succinate (TPGS) compared to TPGS alone [30]. Interestingly, in vitro studies confirmed a protective effect of CoQ10 on RGCs. Similarly, in Adult Dark Agouti rats with unilateral surgically induced ocular hypertension, experiments, using in vivo DARC, and, post-mortem, histological assessment on whole retinal mounts with Brn3a, showed a significant neuroprotective effect of CoQ10/TPGS; these data suggest that topical CoQ10 may be effective in preventing RGC apoptosis and loss in glaucoma [30].

It is well known that oxidative stress triggers the activation of human optic nerve (ONH) astrocytes and upregulates the superoxide dismutase 2 (SOD2) and heme oxygenase-1 (HO-1) protein expression in the ONH astrocytes. Interestingly, in vitro CoQ10 prevented hydrogen peroxide-induced activation of ONH astrocytes, but also significantly decreased SOD2 and HO-1 protein expression in the ONH astrocytes. Thus, preventing mitochondrial mass loss as well as the reduction of cellular ATP [31].

These findings have been confirmed in a study evaluating the results of a diet supplemented with CoQ10 on the oxidative stress and mitochondrial alteration, as well as RGC survival after an ischemic insult of the retina induced by IOP elevation in mice. Interestingly, CoQ10 significantly promoted RGC survival, prevented the upregulation of SOD2 and HO-1 protein expression, and significantly blocked activation of astroglial and microglial cells in ischemic retina [32]. Moreover, the CoQ10 blocked apoptosis by decreasing caspase-3 protein expression, significantly decreased Bax protein expression, and induced pBad protein expression increase in the ischemic retinas. In addition, CoQ10 significantly prevented the increased mitochondrial transcription factor A (Tfam) protein expression at 12 h from the ischemic event. All these data indicate that CoQ10 significantly protects RGCs against oxidative stress by modulating the Bax/Bad-mediated mitochondrial apoptotic pathway as well as prevents mitochondrial alteration by preserving Tfam protein expression [32].

A diet supplemented with CoQ10 was also tested in glaucomatous DBA/2J mice showing a greater RGC survival and preservation of the ONH axons. In addition, astroglial activation was inhibited by decreasing GFAP expression in the retina and ONH of glaucomatous DBA/2J mice. Interestingly, the study reported a significant block of the upregulation of NR1 and NR2A, and a significant block of SOD2 and HO-1 protein expression, as well as a significant prevention of RGC apoptosis, by decreasing Bax protein expression or by increasing pBad protein expression in the retina of glaucomatous DBA/2J mice. More importantly, CoQ10 preserved mitochondrial DNA content and Tfam/oxidative phosphorylation (OXPHOS) complex IV protein expression in the retina of glaucomatous DBA/2J mice. These data suggest that CoQ10 may limit damage by glutamate excitotoxicity and oxidative stress in glaucomatous neurodegeneration [33].

Interestingly, Parisi et al. [34] reported that CoQ10 associated with vitamin E administration in open-angle glaucoma patients has a beneficial effect on the inner retinal function, showed by pattern electroretinogram (PERG) improvement, with consequent enhancement of the visual cortical responses, showed by visual-evoked potential (VEP) improvement [34]. After 6–12 months of treatment, in glaucoma patients treated only with β-blocker, PERG P50 and VEP P100 implicit times were decreased, whereas PERG P50-N95 and VEP N75-P100 amplitudes were increased (*P* < 0.01) when compared to baseline. On the contrary, in the glaucoma group treated with coenzyme Q10 and vitamin E (2 drops per day) in addition to β-blocker monotherapy, the differences in implicit times and amplitudes with respect to baseline were significantly larger (*P* < 0.01) than those recorded in the other group.

Overall, these data suggest a possible usefulness of CoQ10 in addition to the IOP-lowering treatment in glaucoma.

Another promising molecule in the treatment of glaucoma is cytidine 5′-diphosphocholine, also called citicoline. This mononucleotide composed of ribose, cytosine, pyrophosphate, and choline, which is an intermediate endogenous compound in the synthesis of membrane phospholipids [35–37].

This molecule acts stimulating synthesis of phospholipids and phosphatidylcholine, attenuating free fatty acids release, and re-establishing cardiolipin phospholipid component levels of the inner mitochondrial membrane. Citicoline also enhances tyrosine hydroxylase activity and, inhibiting dopamine reuptake increases dopamine levels. Moreover, citicoline increases the levels of other neurotransmitters, such as noradrenaline, serotonin and acetylcholine [38–40].

The protective effects of citicoline in cerebrovascular and neurodegenerative diseases has been extensively discussed in literature. Citicoline has been shown to be effective in reducing infarct volume, brain edema and in improving neurologic deficits in stroke experimental models, as well as in enhancing the level of consciousness after ischemia [41–44]. Citicoline also showed efficacy in Alzheimer’s disease possibly interfering on the deposition of neurotoxic proteins such as β-amyloid, and improving mental performance and brain electrical activity. This was also in part confirmed in a Cochrane systematic review [45]. Moreover, citicoline showed a significant improvement of neurological signs and symptoms such as improvement of rigidity, bradykinesia and tremor in Parkinson’s disease [46–48].

Efficacy of citicoline in glaucoma has also been tested. Experimental studies on rats suggested a possible protective effect on RGCs. Oshitari et al. [49] hypothesized an antiapoptotic effect of citicoline in mitochondria-dependent cell death mechanism, and an auxiliary role in axon regeneration. Using tissue culture of mouse retinal explants, the authors examined the effect of citicoline on damaged RGCs. The TdT-dUTP terminal nick-end labeling-positive cell number in the ganglion cell layer was very low in the retina of mice treated with intraperitoneal citicoline compared to controls. In adult rats with partial optic nerve crush, Schuettauf et al. [50] reported an antiapoptotic effect of citicoline as well as an expression of antiapoptotic protein Bcl-2. In a male rat model of kainic acid-induced retinal damage, the treated group was partially preserved from the gradual retinal thickness reduction found in the control group. Thus, suggesting a neuroprotective effect of citicoline in glutamate-mediated cell death [51].

In a study by Matteucci et al. [52], primary retinal cultures, obtained from rat embryos, were first treated with increasing concentrations of citicoline and analyzed in terms of apoptosis and caspase activation and characterized by immunocytochemistry to identify neuronal and glial cells. Interestingly, citicoline interfered with neuronal cell damage both in glutamate-treated and high glucose-treated retinal cultures by decreasing proapoptotic effects and contrasting synapse loss, thus, confirming the neuroprotective activity in vitro of citicoline.

From a clinical point of view, Pecori-Giraldi et al. [53] reported a stable visual field improvement in glaucoma patients treated with intramuscular citicoline. In a second paper, they also reported that this significant improvement of retinal sensitivity, measured at 1-year follow-up, persisted after 9 years [54].

Using VEP and PERG, Parisi et al. [55] evaluated the effect of a daily intramuscular dose of citicoline on retinal function and on cortical responses in patients with glaucoma. Interestingly, treatment with citicoline induced a significant treatment-dependent improvement of VEP and PERG. This was subsequently confirmed by Parisi [56] who observed, in a 8-year follow-up study, a significantly improved retinal and cortical responses in glaucoma patients following administration of citicoline. These observations suggest a potential use of citicoline as a complement to IOP-lowering medications in the treatment of glaucoma.

The group of Rejadak et al. [57] was the first to test the efficacy of citicoline tablets using VEP. Interestingly, after treatment VEP latency was reduced and VEP amplitude was increased in a large percentage of the treated eyes, thus suggesting that oral citicoline improves VEPs in glaucoma patients.

In 2008, citicoline oral suspension and intramuscular citicoline effects were compared using VEP and PERG showing no differences among the two types of route of administration [58]. A multicenter study subsequently showed a reduction of the rate of progression of visual field in patients with progressive glaucoma after treatment with citicoline oral solution [59], suggesting that citicoline oral solution formulation bioavailability can be as good as the parenteral administration [60, 61].

The next step was the evaluation of the use of topical citicoline eye drops. Experimental study showed citicoline presence in the mice vitreous after its administration in a topical solution with benzalkonium chloride and hyaluronic acid [62]. A subsequent clinical phase, using visual field indices and electrofunctional tests, evaluated glaucoma progression in 16 affected patients treated with topical citicoline in association with IOP-lowering agents and in 18 patients treated only with hypotensive agents. This study showed that citicoline eye drops seem to have a neuroprotective effect that is independent of IOP-lowering effect due to hypotensive drops. In this regard, visual field indices showed a positive trend in individual eyes of patients treated with topical citicoline in association with IOP-lowering agents, but these values were not statistically significant in the whole group. Moreover, PERG showed reduced P50 latency (*P* = 0.04) and increased P50-N95 amplitude (*P* < 0.0001), suggesting an RGC function improvement. This improvement was still present after 30 days of discontinuing treatment [62].

Recently, Parisi et al. [63] in a prospective, randomized, study confirmed that administration of citicoline eye drops three times a day significantly improved PERG and VEP respect to controls and baseline. In particular, patients who displayed the worst retinal function at baseline showed greater benefits from citicoline eye drops treatment. This improvement, evaluated using electrophysiological parameters, correlated with visual field mean deviation [63].

Recently, citicoline has been approved in the European Union market as a novel food ingredient in food supplements and in dietary foods for special medical purposes (2014/423/EU). Moreover, the Italian Ministry of Health has approved a product with citicoline in oral solution as dietary food for special medical purposes with the therapeutic indication for glaucomatous patients pharmacologically stabilized but with progressive loss of visual field.

Other molecules with neuroprotective potential are Ginkgo biloba and palmitoylethanolamide (PEA). Ginkgo biloba has been known for hundreds of years and its extracts mainly consist of flavonoids and terpenoids. Most of the literature has evaluated the pharmacological effects of two main extracts of this substance: EGb 761 and LI 1370 [64]. Owing to its hemorheological and antioxidant properties, ginkgo biloba has a stabilizing effect on microcirculation and mitochondrial membranes, respectively. Since numerous mitochondrial abnormalities have been implicated in glaucoma pathogenesis, it has been hypothesized that this molecule may play a neuroprotective action in that pathology [65, 66]. Finally, recent evidence has revealed involvement of the endocannabinoid system in the pathogenesis of many neurodegenerative diseases and studies have indicated that this system is also involved in the control of IOP [67]. PEA was tested in at least nine double blind studies, two of which were on a glaucoma model and was considered safe and effective [68]. The administration of PEA, inhibiting various inflammatory cascades that appear to be relevant in glaucoma, has shown the ability to reduce IOP and improve visual field indices in glaucoma subjects with normal tension glaucoma, thus suggesting a dual, IOP-lowering and neuroprotective, action [69, 70].

## Conclusion

It is widely accepted that glaucoma is a multifactorial ocular disease, it is therefore conceivable that a variety of treatments, in addition to conventional IOP-lowering therapies, may be helpful to interfere with progression of the disease. Recently, several molecules have been evaluated in the treatment of glaucoma, although most of them failed in the clinical trial. This is probably due to the fact that the animal models do not accurately simulate the human disease or that its variability in humans is higher than in laboratory animals. Moreover, to establish the correct endpoint of a clinical trial for neuroprotection is a complex process. Visual function is among the most important; however, to date there is no optimum way to measure it. Most of the studies are based on functional tests that are not always accurate. Therefore, it is conceivable that developments in electrophysiological, perimetric, and imaging techniques will give a boost to the research on neuroprotectants, helping to confirm the usefulness of current available molecules or helping novel drugs to emerge.
